# Preclinical studies of targeted *α* therapy for breast cancer using ^213^Bi-labelled-plasminogen activator inhibitor type 2

**DOI:** 10.1038/sj.bjc.6600838

**Published:** 2003-03-18

**Authors:** B J Allen, Z Tian, S M A Rizvi, Y Li, M Ranson

**Affiliations:** 1 Centre for Experimental Radiation Oncology, St George Cancer Care Centre, Gray St., Kogarah, NSW 2217, Australia; 2 University of NSW, Kensington, NSW 2052, Australia; 3 University of Wollongong, NSW 2522, Australia

**Keywords:** breast cancer, MDA-MB-231 cell line, targeted *α* therapy, *α*-particle emitter ^213^Bi, plasminogen activation inhibitor type 2

## Abstract

The control of micrometastatic breast cancer remains problematic. To this end, we are developing a new adjuvant therapy based on ^213^Bi-PAI2, in which an *α*-emitting nuclide (^213^Bi) is chelated to the plasminogen activator inhibitor-2 (PAI2). PAI2 targets the cell-surface receptor bound urokinase plasminogen activator (uPA), which is involved with the metastatic spread of cancer cells. We have successfully labelled and tested recombinant human PAI2 with the *α* radioisotope ^213^Bi to produce ^213^Bi-PAI2, which is highly cytotoxic towards breast cancer cell lines. In this study, the 2-day postinoculation model, using MDA-MB-231 breast cancer cells, was shown to be representative of micrometastatic disease. Our *in vivo* efficacy experiments show that a single local injection of ^213^Bi-PAI2 can completely inhibit the growth of tumour at 2 days postcell inoculation, and a single systemic (i.p.) administration at 2 days causes tumour growth inhibition in a dose-dependent manner. The specific role of uPA as the target for ^213^Bi-PAI2 therapy was determined by PAI2 pretreatment blocking studies. *In vivo* toxicity studies in nude mice indicate that up to 100 *μ*Ci of ^213^Bi-PAI2 is well tolerated. Thus, ^213^Bi-PAI2 is successful in targeting isolated breast cancer cells and preangiogenic cell clusters. These results indicate the promising potential of ^213^Bi-PAI2 as a novel therapeutic agent for micrometastatic breast cancer.

The major failure in the management of early breast cancer is the incomplete killing of malignant cancer cells that have spread throughout the body (Allen, 1999a). This is despite the many treatments available, such as surgery, radiation therapy, hormone therapy and chemotherapy. The American Cancer Society (2001) estimated 182 800 new cases of invasive breast cancer in the year 2000 among women in America, and 40 800 are expected to die from the disease (American Cancer Society, 2000). Novel, more effective treatments that overcome this problem in breast cancer management are essential. Targeted therapy, first discussed over 100 years ago, is based on the idea that a drug will attack its target without damaging other tissue (Raso, 1990). Targeted alpha therapy (TAT) uses an *α*-emitting radionuclide as a lethal medicament via an effective targeting carrier to kill cancer cells (McDevitt *et al*, 1998; Allen, 1999b). We are investigating a novel targeting approach that exploits the involvement of cell-surface receptor bound urokinase plasminogen activator (uPA) in the metastatic spread of breast cancer cells (Kruithof *et al*, 1995).

*α*-emitting radionuclides emit *α* particles with energies of 4–8 MeV, which are up to an order of magnitude greater than most *β* rays. Yet, their ranges are two orders of magnitude less as *α* particles have a linear energy transfer (LET) which is about 100 times greater (Allen, 1999a). This is manifested by a higher relative biological effectiveness (RBE). As a result, a much greater fraction of the total energy is deposited in cells with *α*'s and very few nuclear hits are required to kill a cell. Consequently, only *α* radiation has the potential to kill the metastatic cancer cells at tolerable dose limits, whereas the low LET of *β*'s makes this a very difficult task within human dose tolerance limits.

Availability of the *α*-emitting radionuclides has been the major problem in the past for their large-scale scientific and clinical application. Studies have been carried out using ^149^Tb (Allen, 1999a; Rizvi *et al*, 2001), ^211^At (Bloomer *et al*, 1984; Larsen *et al*, 1998) and ^212^Bi (Macklis *et al*, 1993; Horak *et al*, 1997) with encouraging results. The stable and reliable ^225^Ac generator of the *α* emitting nuclide ^213^Bi has been produced, modified and used successfully, with several of these studies indicating a therapeutic potential of ^213^Bi-labelled antibody constructs against cancer cells both *in vitro* and *in vivo* (Van Geel *et al*, 1994; Boll *et al*, 1998; Kennel, 1999a,1999b; McDevitt *et al*, 1999; Nikula *et al*, 1999; Adams *et al*, 2000; McDevitt *et al*, 2000). Our group has modified methods of conjugating ^213^Bi radionuclide to antibodies with the stable chelator cyclic diethylenetriaminepentacetic acid anhydride (cDTPA) for use in the *α* therapy of melanoma (Rizvi *et al*, 2000; Allen *et al*, 2001a,2001b), colorectal cancer (Rizvi *et al*, 2001), leukaemia (Rizvi *et al*, 2002), breast (Ranson *et al*, 2002) and prostate cancer (Li *et al*, 2002a,2002b).

A large body of experimental and clinical evidence implicates overexpression of the urokinase plasminogen activator (uPA) system as a modulator of the aggressive behaviour of cancer cells and as a strong prognostic factor for predicting poor breast cancer patient outcome (Pollanen *et al*, 1991; Andreasen *et al*, 1997; Schmitt *et al*, 2000). uPA converts plasminogen into the highly active protease plasmin. Plasmin promotes tissue degradation and remodelling of the local extracellular environment by directly and indirectly (via activation of prometalloproteases) degrading extracellular matrix molecules. uPA is synthesised and secreted as a proenzyme, whose activation is markedly accelerated upon binding with high affinity (0.1–1 nM) to specific cell-surface uPA receptors (uPAR) (Pollanen *et al*, 1991; Schmitt *et al*, 1992). Receptor density varies depending on cell type (10^3^–10^6^ sites cell^−1^; Schmitt *et al*, 1992). The ability of PAI2 to inhibit tumour invasion and metastases in animal models has been demonstrated by several laboratories utilising uPA-overexpressing cancer cells (Ranson *et al*, 2002).

PAI1 conjugated to A-chain cholera toxin as the cytotoxic agent or modified PAI1 conjugated to saporin has been used to target fibrosarcoma cells (Jankun, 1992,^1994^) with moderate cytotoxicity. However, PAI2 has several distinct advantages over PAI1 for targeted cancer therapy, as discussed in Ranson *et al* (2002). Cell surface-bound uPA is accessible to and inhibitable by exogenous PAI2 (Jankun, 1992; Yang *et al*, 2000), and a number of studies have suggested the potential for PAI2 to inhibit cancer cell invasion and metastasis (Kruithof *et al*, 1995).

The pharmacokinetics and biodistribution of human recombinant ^125^I-labelled PAI2 in both control mice and mice bearing human colon cancer (uPA-positive HCT116 cell line) xenografts have been established (Hang *et al*, 1998). Such studies indicate that invasive and metastatic tumour cells, shown consistently to contain active uPA, would be accessible to and targeted by exogenously administered PAI2.

It is clear that uPA is a specific marker of malignancy and that PAI2 represents a useful targeting agent. We have previously reported the production and evaluation of the new *α*-nuclide emitting cytotoxic agent ^213^Bi-labelled PAI2 (Ranson *et al*, 2002). The reactivity, specificity and cytotoxicity of *α*-PAI2 were reported for both MDA-MB-231 and MCF-7 human breast cancer cell lines *in vitro*. Immunohistochemistry mirrored the differences in expression of endogenous uPA and uPAR antigen seen in these two cell lines by flow cytometry (Ranson *et al*, 2002).

We have also carried out *in vitro* and *in vivo* studies of ^213^Bi-PAI2 for prostate cancer (Li *et al*, 2002b), finding it to be efficacious within the maximum tolerance dose limits. We now demonstrate the efficacy of TAT with ^213^Bi-PAI2 in a nude mouse breast cancer model, using MDA-MB-231 breast cancer cells. These data clearly show that ^213^Bi-PAI2 has an important role as a potential new therapeutic modality for the control of micrometastases in breast cancer.

## MATERIALS AND METHODS

Human recombinant PAI2 (47 kDa) was provided by Biotech Australia Pty Ltd RPMI-1640 was purchased from Life Technologies (Castle Hill, NSW, Australia). Fetal calf serum (FCS) was obtained from Trace Bioscientific (Castle Hill, NSW, Australia). The cyclic anhydride of diethylenetriaminepentacetic acid (cDTPA) was purchased from Aldrich Chemical Company, bovine serum albumin (fraction V) (BSA) from Sigma Chemical (St Louis, MO, USA), and mouse anti-human uPA IgG_1_ (#394) monoclonal antibody (MAb) from American Diagnostica Inc. (Greenwich, CT, USA). Mouse isotype control subclasses IgG_1_ MAb was from Silenus (Sydney, NSW, Australia) and rabbit anti-mouse IgG and alkaline phosphatase and anti-alkaline phosphatase (APAAP) were purchased from Dakopatts (Glostrap, Denmark). Mouse anti-human melanoma IgG2a MAb (9.2.27) used as a nonspecific control was kindly provided by the Royal Newcastle Hospital (Sydney, Australia).

### Radioisotope

*α*-particle-emitting radionuclide ^213^Bi was produced from the ^225^Ac/^213^Bi generator, purchased from the United States Department of Energy, Oak Ridge National Laboratory (Oak Ridge, TN, USA). ^213^Bi was eluted from the ^225^Ac column with 250 *μ*L of freshly prepared 0.15 M distilled and stabilised hydriodic acid followed by washing with 250 *μ*L sterile distilled water (Boll *et al*, 1997). The first elution was not used, and a time of 2 h was allowed for ^213^Bi to regenerate on the column for the next elution. Activity corrections were made for ^213^Bi decay using the half-life of 46 min.

### PAI2 conjugation with cDTPA, stoichiometry and reactivity

PAI2 and BSA were conjugated with cDTPA by a modification of published methods to give the desired protein-DTTA conjugate (Ranson *et al*, 2002) while MAb 9.2.27 was conjugated with cDTPA as described previously (Rizvi *et al*, 2000) for nonspecific control.

The concentrations of the protein-DTTA chelates were measured by BIORAD DC protein assay reagent kit (Pierce, Rockford Il, USA). The stoichiometry of DTTA-PAI2 was determined using electrospray ionisation mass spectrometry as previously described (Ranson *et al*, 2002). The DTTA-PAI2 was diluted in 1:20 in water and then 1:2 with 50% MeOH and 1% acetic acid.

### ^213^Bi labelling of DTTA-PAI2

Concentrated DTTA-PAI2 stocks were diluted with 500 mM sodium acetate at pH 5.5 and 5–10 *μ*g of DTTA-PAI2 was labelled with free ^213^Bi for 20 min at room temperature as described previously (Ranson *et al*, 2002). The radiolabelling efficiency was about 90%, as determined by instant thin layer chromatography (ITLC) using the described method (Rizvi *et al*, 2000; Ranson *et al*, 2002).

### Serum stability study

^213^Bi-PAI2 was incubated in fresh human serum and in DTPA (challenge test) at 37°C over 24 h. Activity was counted by detecting the 440 keV gamma ray from the decay of ^213^Bi. The percentage leaching/stability was calculated as a function of time, and the results were corrected for decay of the radioisotope.

### Cell culture

The metastatic MDA-MB-231 human breast cancer cell line was originally purchased from American Type Culture Collection (Rockville, MD, USA) and routinely cultured in RPMI-1640 supplemented with 10% (v v) heat-inactivated FCS and passaged using trypsin/EDTA. The cells were incubated in a humidified incubator at 37°C with a 5% carbon dioxide air atmosphere. For all experimental procedures, subconfluent cells that had been in culture for 48 h without a change of media were harvested by rinsing flasks twice with PBS (pH 7.2) and then detaching with PBS/0.5 mM EDTA at 37°C for 5 min. Cells were collected and resuspended in the appropriate buffer as described below.

### Animals and MDA-MB-231 cell inoculation

In all, 6 to 8-week-old athymic nude mice, BALB/c (nu/nu) female mice were purchased from Animal Resources Centre (ACR), Western Australia. The mice were housed and maintained in laminar flow cabinets under specific pathogen-free conditions in facilities approved by the University of New South Wales (UNSW) Animal Care and Ethics Committee (ACEC) and in accordance with their regulations and standards. The ethical guidelines that were followed meet the standards required by the UK Coordinating Committee on Cancer Research Guidelines (Workman *et al*, 1998).

To establish s.c. animal tumour models, 1 million MDA-MB-231 cells were resuspended in 200 *μ*L of RPMI-164 serum-free medium and injected via an 18-gauge needle into bilateral mammary fat pads of each nude mouse. Tumour progression was documented once weekly by measurements using calipers, and tumour volumes were calculated by the following formula: length × width × height × 0.52 in millimetres (Gleave *et al*, 1991). All mice were killed when the xenografts approached 1 × 1 cm^2^ in area by CO_2_ chamber.

Control injections used PBS and the nonspecific *α*-conjugates ^213^Bi-BSA and ^213^Bi-9.2.27 (monoclonal antibody for melanoma).

### Experimental protocols

*In vivo toxicity study*: Groups of five mice received a total injected activity (bound plus unbound ^213^Bi) of 1.5, 3 and 6 mCi/kg weight dose of ^213^Bi-PAI2 by i.p. injection. Additional mice were treated with PAI2, cDTPA and PBS as controls. Mice were monitored and weight was measured.

*Biodistribution study*: Mice received an i.p injection of ^213^Bi-PAI2, and were euthanised at 15, 30, 45, 60, 90 and 120 min. Tissues and organs were removed, weighed and the activity was counted. The bone marrow count was obtained by measuring activity in the hip. Results are quoted as percent activity at a given euthanasia time.

*Two-day MDA-MB-231 model*: One million cells were injected s.c. into bilateral mammary glands of five mice, and at 2 days the mice were killed and the local tissue section removed for histochemistry. The objective of this study was to demonstrate the state of pretumour development.

*Local TAT efficacy for dose response*: Efficacy studies of local TAT were made for dose response and for postinoculation time response at 2 days postinoculation. The ^213^Bi-PAI2 was injected in the same region as the inoculation, as no tumour was evident.

*Local TAT efficacy for time response:* Four different therapy time points were used, each with five mice: 2–4, 7, 14 and 28 days after cell inoculation. Each group had one control mouse and four treated mice.

*Systemic TAT with* ^*213*^*Bi-PAI2*: The dose response for systemic administration (i.p.) injection at 2 days postinoculation was also studied. Previous studies had shown little difference between i.p. and tail vein injections, so the more difficult tail vein approach was not justified.

*PAI2 blocking study:* At 2 days postbilateral inoculation each of 10^6^ cancer cells, i.p. administration at two, three and four times the standard PAI2 conjugate concentration (100 *μ*g mL^−1^) was followed by 100 *μ*Ci of ^213^Bi-PAI2 in groups of five mice. Tumour growth was monitored in all mice and mice were killed at the tumour volume limit.

### Statistics

All numerical data were expressed as the average of the values obtained, and the standard error of means (s.e.m.) was calculated and shown in the figures. Unpaired *t*-test was used to determine significant differences at 0.05 probability between tumour growth expressed as volume for controls and ^213^Bi-PAI2-treated mice.

### Immunohistochemistry

The alkaline phosphatase antialkaline phosphate (APAAP) method was used to detect uPA expression in MDA-MB-231 cells after 2 days inoculation in nude mice (Li *et al*, 2002b). Control slides were treated in an identical manner. PC3 metastatic prostate cancer cells were chosen as a positive control, while isotype MAb or the primary antibody were omitted as a negative control. The positive cells appear pink.

## RESULTS

### Chelation of PAI2 with cDTPA

**Figure 1:**
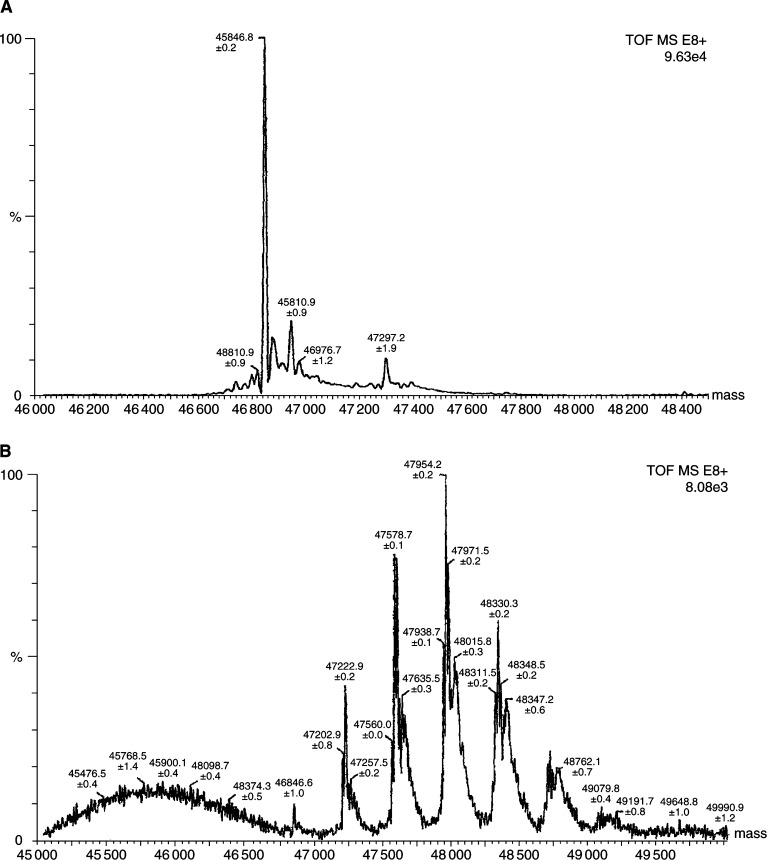
Chelation of PAI2 with cDTPA. Mass spectroscopy results for PAI2 (**A**) and DTTA-PAI2 (**B**). Multiple attachment of the chelator occurs, as is evident by the peaks shifted by the MW=357 Da of the chelator cDTPA.

Mass spectroscopy results are shown in PAI2 alone (Figure 1A

) and DTTA-PAI2 (Figure 1B). Up to five-fold attachment of cDTPA is observed, the peaks being separated by the MW of the chelator (357 Da).

### Serum stability test

Most of the instability of ^213^Bi-PAI2 in serum and in DTPA (challenge test) occurs within one half-life of ^213^Bi (data not shown). Leaching of activity is in the range from 20 to 30%. Curiously, the conjugate is more stable in the DTPA challenge.

### *In vivo* tolerance study

The weights of injected mice reduced initially by 5–10%, then recovered after 1 week. After 13 weeks, one saline control mouse died, but other mice were healthy until euthanasia at 24 weeks post-therapy (data not shown). No dose effect was observed.

### Biodistribution

**Figure 2:**
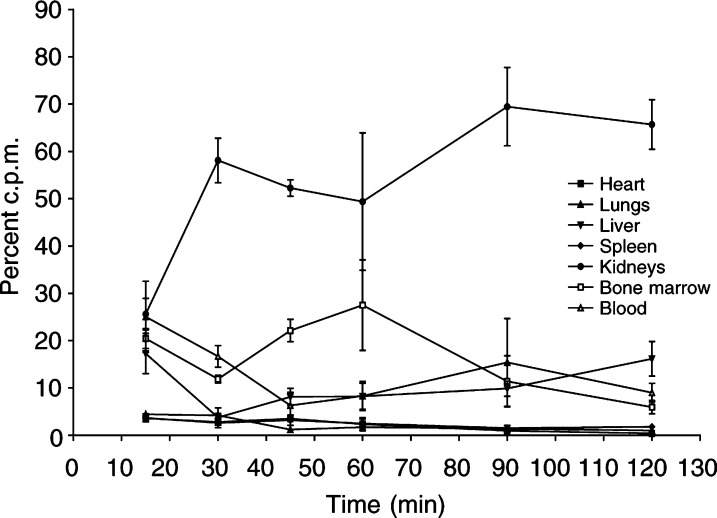
Biodistribution study of ^213^Bi-PAI2 in nude mice. The kidneys receive more than 50% of the radiation exposure after 30 min.

Results were obtained over 2.6 half-lives (Figure 2

) and showed that the kidneys received the highest activity, being more than half the observed activity after 25 min. The bone marrow receives the next highest dose, but other organs have relatively low exposure.

### Expression of uPA *in vivo* model after 2 days inoculation

**Figure 3:**
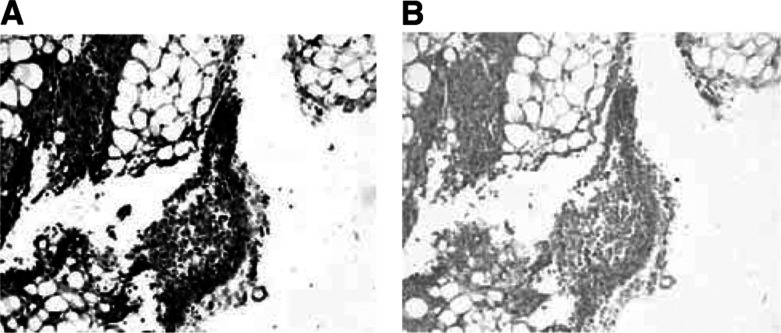
uPA expression in 2 days MDA-MB-231 breast cancer cells inoculation model sections. The sections stained with MAb #394 are positive to uPA Mab (**A**) while the control sections with no primary antibody are negative to uPA MAb light grey (**B**). The dark grey colour indicates positive cancer cells. Isolated cells and cell clusters are apparent, with no evidence of capillary growth. Magnification: A, B × 100.

A group of five mice were sacrificed at 2 days after cell inoculation. The results from immunostaining with the #394 MAb against uPA show that isolated cells and cell clusters are prevalent, all cancer cells are positive to uPA, and there is no evidence for microcapillary formation (Figure 3A

) while the cancer cells with no primary MAb are negative to uPA MAb (Figure 3B). Thus the 2-day model accurately simulates micrometastasis and preangiogenic lesions.

### Tumour growth inhibition by local injection of ^213^Bi-PAI2

In an earlier study (Allen *et al*, 2001a), local injection at 2 days postinoculation of 12, 25 and 50 *μ*Ci of ^213^Bi-PAI2 showed a dose-dependent response in groups of five mice. Tumours grew quickly after injection with a PBS control, while the 12 *μ*Ci group grew very slowly. The 25 and 50 *μ*Ci groups showed complete inhibition of tumour growth.

**Figure 4:**
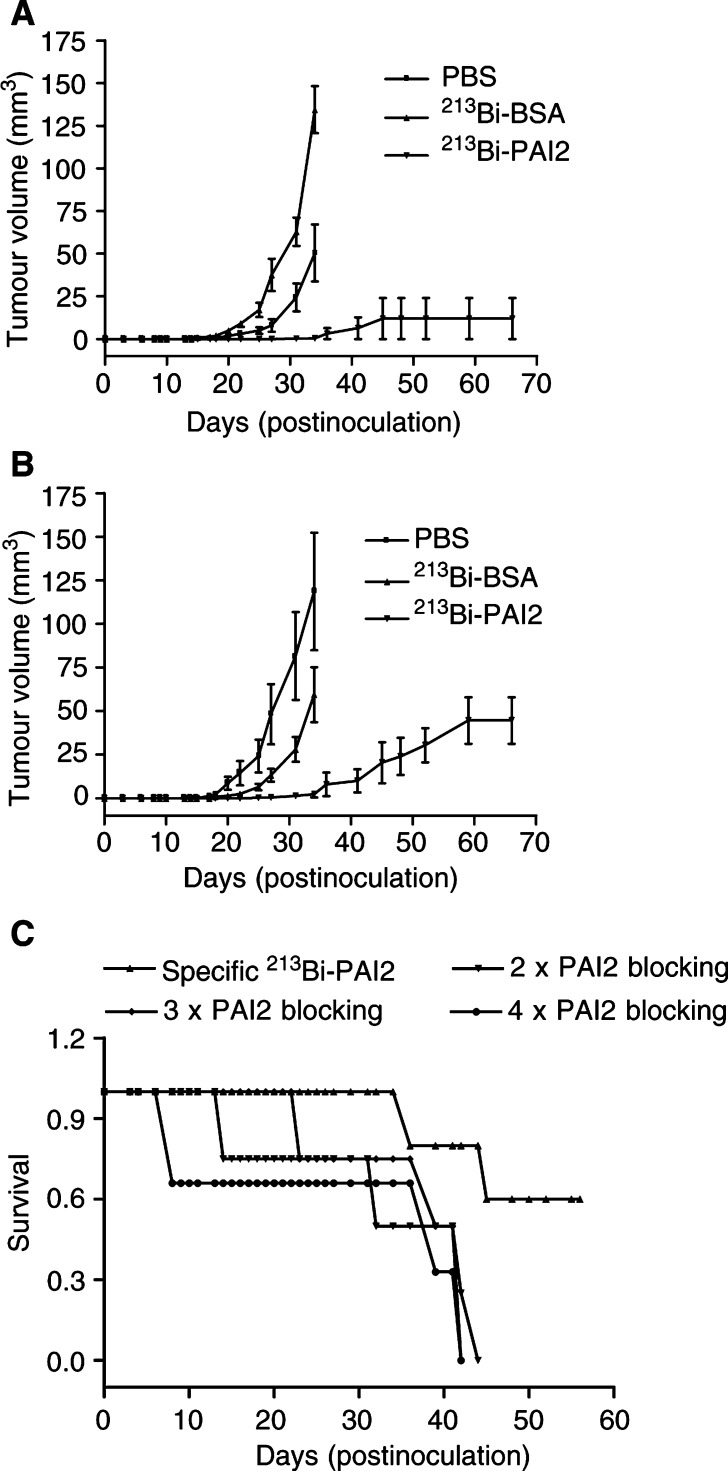
The effect of a single dose of ^213^Bi-PAI2 on tumour growth of s.c. 2 days MDA-MB-231 breast cancer cells in nude mice with local and i.p. injection in mice and the PAI2 blocking experiment. (**A**) Bilateral single local injections each of 50 *μ*Ci ^213^Bi-PAI2(▾) or 50 *μ*Ci ^213^Bi-BSA (▴) and the same volume of PBS buffer (▪). (**B**) 100 *μ*Ci i.p. administration of ^213^Bi-PAI2 (▾) or the same activity of ^213^Bi-BSA (▴) and the same volume of PBS buffer (▪). (**C**) Pre-injection of PAI2, followed by 100 *μ*Ci of ^213^Bi-PAI2, allows all tumours to grow to the terminating end point at 30–40 days. ^213^Bi-PAI2 alone inhibits the growth of eight out of 10 tumours to 60 days (▴) while the ‘blocked’ mice with 2 × PAI2 (▾), 3 × PAI2 (⧫) and 4 × PAI2 (•) can only survive up to 40 days. This blocking study shows that uPA receptor sites on the MDA-MB-231 cells are saturated at twice the therapeutic PAI2 dose of 100 *μ*g ml^−1^. No death related to toxicity occurred. Data are expressed as the mean±s.e.m. for five tumours in each case.

This study was repeated using bilateral injections of 50 *μ*Ci of ^213^Bi-PAI2 and ^213^Bi-BSA (which has comparable mass to ^213^Bi-PAI2) and PBS as controls. Results are shown in Figure 4A

. Both PBS and ^213^Bi-BSA controls are significantly different to the ^213^Bi-PAI2 results (*P*=0.04 and 0.03, respectively).

A single injection of ^213^Bi-PAI2 (25 *μ*Ci) was made into cell inoculation sites or tumours at different postinoculation times. Mice were monitored and tumours were measured. The 2–4-day group had the best response to the therapy, which had some 50% (23 out of 40) tumour control and slower tumour growth rate compared with control mice. The 7-day and 14-day groups had two out of eight and one out of eight tumour control and slower growth rate compared with control mice. The 28-day group had zero out of eight tumour control and no obvious change in tumour growth rate.

### Efficacy of ^213^Bi-PAI2 by systemic injection

**Figure 5:**
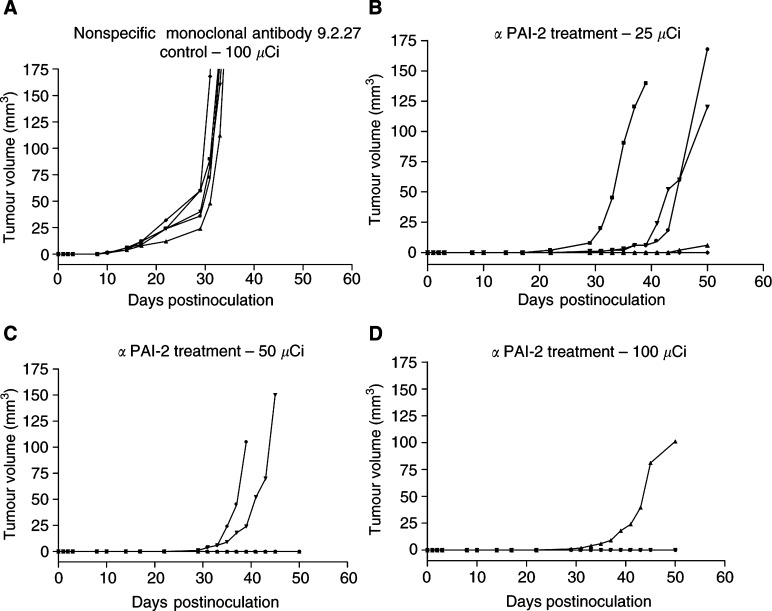
Effect of a single i.p. injection of ^213^Bi-PAI2 with different activities on tumour growth in the 2-day MDA-MB-231 nude mice model. (**A**) 100 *μ*Ci of nonspecific ^213^Bi-9.2.27. (**B**) 25 *μ*Ci of ^213^Bi-PAI2. (**C**) 50 *μ*Ci ^213^Bi-PAI2. (**D**) 100 *μ*Ci ^213^Bi-PAI2. A dose response is indicated, with eight out of 10 tumours being controlled at 100 *μ*Ci of ^213^Bi -PAI2.

Mice received single systemic (i.p.) injections of 25, 50 and 100 *μ*Ci of ^213^Bi-PAI2 at 2 days postinoculation. The results indicate a substantial inhibition of tumour growth up to 35 days postinoculation. The control mice (*n*=5) received a single i.p. injection of 100 *μ*Ci nonspecific ^213^Bi-9.2.27 conjugate, which had no effect on tumour growth (Figure 5A

), while in the treated groups (*n*=5), a dose effect is indicated in that increased activity decreased the number of observed tumours, that is, three out of five in 25 *μ*Ci (Figure 5B), two out of five in 50 *μ*Ci (Figure 5C) and one out of five in 100 *μ*Ci (Figure 5D). The control data are significantly different from the ^213^Bi-PAI2 data for all dose levels.

A second study was made with both PBS and ^213^Bi-BSA controls, and the results are shown in Figure 4B. The controls are not significantly different from each other, but the PBS and ^213^Bi-BSA controls are significantly different from the ^213^Bi-PAI2 result (*P*=0.01 and 0.04, respectively). Note that only a single i.p. injection of 100 *μ*Ci was made.

### PAI2 blocking study

Systemic (i.p.) injection of PAI2 at two, three and four times ^213^Bi-PAI2 was administered, followed by the ^213^Bi-PAI2 conjugate. Results are shown in Figure 4C, where tumours grew in all ‘PAI2 blocked’ mice, whereas eight out of 10 ‘unblocked’ mice showed complete tumour growth inhibition to 60 days. Pretreated mice showed tumour volumes that were not significantly different for each concentration. However, they were all significantly different to the ‘unblocked’ mice (*P*<0.001). The ‘blocked’ mice were euthanised at 30–45 days according to protocol. All excess concentrations of PAI2 were adequate to effectively block MDA-MB-231 breast cancer cells receptors at 2 days postbilateral inoculation.

## DISCUSSION

In this study, we describe the novel compound ^213^Bi-PAI2 and show that it retains reactivity, selectivity and cytotoxicity towards uPA expressing breast cancer cells *in vivo*. That ^213^Bi-PAI2 cytotoxicity is significantly mediated via a uPA-dependent mechanism had been confirmed by the lack of cytotoxicity of freshly isolated, normal human leukocytes on which cell-surface localised active uPA was not detectable (Ranson *et al*, 2002). ^213^Bi-PAI2 was found to be selectively toxic to targeted breast cancer cells, whereas nontargeted cells are spared from the radiotoxicity arising from the *α* radiation. Breast cancer cells incubated with a nonspecific *α*-conjugate, viz ^213^Bi-BSA, were also minimally affected.

Mass spectroscopy analyses show that multiple binding of the cDTPA chelator occurs. While this will enhance the activity carried by PAI2, it may cause problems for quality control of specific activity. The stability of the *α*-conjugate is also a cause for concern. Some 20% of activity is lost in serum at 37°C within 1 half-life. More stable conjugation is desired.

Minimal toxicity of ^213^Bi-PAI2 was observed in mice up to a total activity of 6 mCi kg^−1^. This dose level is expected to be more than adequate for human use, and compares with 1 mCi kg^−1^ reported for ^213^Bi-MAb in a phase 1 clinical trial by the Memorial group (Jurcic *et al*, 2002).

The biodistribution of ^213^Bi-PAI2 in mice showed that the kidneys received the highest dose (>50% after 25 min). Further, the kidneys continued to accumulate activity up to 100 min. This result may have implications for the induction of secondary renal cancer at higher doses.

Resection and histopathology of the 2-day postinoculation breast cancer model showed abundant isolated cells and clusters of cancer cells at the inoculation site, but no evidence for capillary formation was found. This model is therefore expected to simulate micrometastatic cancer. Our *in vivo* studies revealed that ^213^Bi-PAI2 can target isolated cells and preangiogenic cell clusters. Local therapy required only 25 *μ*Ci of *α*-PAI2 to achieve complete inhibition of tumorigenesis. ^213^Bi-PAI2 was found to be increasingly less effective in a single dose intralesional protocol as the tumours grew in size.

A higher dose of 100 *μ*Ci was required for a single systemic administration to achieve ∼80% inhibition, and indications of a dose-dependent response were seen. All control results were found to be significantly different from the ^213^Bi-PAI2 data. The specific, *in vivo* cytotoxicity of ^213^Bi-PAI2 against the uPA receptor was directly tested by preinjection of increasing concentrations of PAI2, before ^213^Bi-PAI2 administration. Tumours grew in all ‘blocked’ mice, which were euthanised according to protocol at 30–40 days, whereas only two out of 10 tumours grew in the unblocked ^213^Bi-PAI2 mice.

Our results clearly indicate that PAI2 can target membrane-bound uPA receptors, deliver *α* particles to MDA-MB-231 metastatic breast cancer cells and regress tumour growth through local or systemic injections. The exact mechanism of cell killing has not been investigated. Macklis *et al* (1992) demonstrated that *α*-particle radioimmunotherapy can induce apoptosis in murine lymphoma cells. Using the ^213^Bi-PAI2 conjugate, we have also demonstrated that ^213^Bi-PAI2 can induce a high percentage of TUNEL-positive PC3 prostate cancer cells *in vitro* and *in vivo* (Li *et al*, 2002b). These data suggest that apoptosis may be the lethal pathway of ^213^Bi-PAI2 therapy for MDA-MB-231 breast cancer cells.

## CONCLUSIONS

We have combined the cytotoxicity of an *α*-emitting radioisotope (^213^Bi) with the targeting potential of PAI2 to create the novel construct ^213^Bi-PAI2, a potential new therapeutic agent for targeted *α* therapy of breast and prostate cancer (Li *et al*, 2002b). The *in vitro* cytotoxicity of ^213^Bi-PAI2 on breast cancer cells was shown to be specific, indicating that the cell killing ability of ^213^Bi-PAI2 depends critically on the targeting of cells in a receptor-bound, active uPA-dependent manner (Ranson *et al*, 2002). These *in vivo* results show conclusively that ^213^Bi-PAI2 can target and kill isolated cells and cell clusters, via local and systemic injection. Further, as tumours in mice pretreated with PAI2 could not be regressed, targeting of uPA/uPAR is implicated. ^213^Bi-PAI2 therapy is therefore indicated for the control of micrometastic breast cancer.
